# Targeting L‐Selectin Lymphocytes to Deliver Immunosuppressive Drug in Lymph Nodes for Durable Multiple Sclerosis Treatment

**DOI:** 10.1002/advs.202300738

**Published:** 2023-05-12

**Authors:** Yipeng Zhao, Jie Zhang, Xi Cheng, Wenping Huang, Shishi Shen, Shilin Wu, Yiying Huang, Guangjun Nie, Hai Wang, Wei Qiu

**Affiliations:** ^1^ Department of Neurology The Third Affiliated Hospital of Sun Yat‐sen University Guangzhou 510000 China; ^2^ CAS Key Laboratory for Biomedical Effects of Nanomaterials & Nanosafety CAS Center for Excellence in Nanoscience National Center for Nanoscience and Technology Beijing 100190 China; ^3^ School of Nanoscience and Engineering University of Chinese Academy of Sciences 100049 Beijing China

**Keywords:** CD4^+^ T lymphocytes, CD47, FTY720, ketogenic diet, L‐selectin, lymph nodes, multiple sclerosis

## Abstract

Inflammation induced by autoreactive CD4^+^ T lymphocytes is a major factor in the pathogenesis of multiple sclerosis (MS). Immunosuppressive drugs, such as FTY720, are subsequently developed to prevent the migration of CD4^+^ T lymphocytes to the central nervous system (CNS). However, these immunosuppressive drugs have limited accumulation in lymph nodes (LNs), resulting in poor efficacy. Here, this work develops a nanoplatform for delivering immunosuppressive drugs to LNs for durable MS treatment. Human CD47 peptide and L‐selectin targeting aptamer are modified on the nanoparticles encapsulated with FTY720 (clnFTY) for self‐passivation and the targeting of L‐selectin on lymphocytes, a homing receptor for T‐cells entering LNs. Using this natural process, clnFTY nanoparticles efficiently deliver FTY720 to LNs and delay disease progression in experimental autoimmune encephalomyelitis (EAE) mice following a single dose treatment over a 42‐day observational period. Considering the daily dosing requirement of FTY720, this strategy greatly improves its therapeutic efficiency. The ability of clnFTY nanoparticles to target lymphocytes, reduce sphingosine‐1‐phosphate receptor 1 (S1PR1) expression, and suppress inflammatory cytokines release are demonstrated in clinical blood samples from MS patients. Taken together, this study demonstrates that targeted LNs delivery may greatly extend the treatment cycle of immunosuppressive drugs for durable MS treatment.

## Graphical abstract

Liposomes are used to deliver FTY720 by self‐assembling them in lipid membranes and decorating L‐selectin targeting aptamers to target L‐selectin expressing lymphocytes for lymph nodes (LNs) delivery. Owing to efficient delivery of FTY720 in LNs, nanodrugs efficiently inhibit lymphocytes infiltration in central nervous system (CNS) and alleviate injury in the experimental autoimmune encephalomyelitis (EAE) mice.

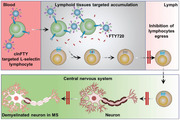

## 1 Introduction

Multiple sclerosis (MS) is a classic central nervous system (CNS) autoimmune disease that affects millions of people worldwide.^[^ ^1^ ^]^ Currently, the cause of MS remains unknown. In experimental autoimmune encephalomyelitis (EAE), autoreactive immune cells infiltrate the spinal cord and brain from the periphery, leading to inflammatory demyelination and white matter injury.^[^ ^2^ ^]^ CD4^+^ T lymphocytes are thought to be primarily responsible for these processes in MS.^[^ ^3^ ^]^ Upon activation in the lymph nodes (LNs), CD4^+^ type 1 T helper (Th1) cells and type 17 T helper (Th17) cells disrupt the blood‐brain barrier (BBB) and trigger CNS inflammation by releasing inflammatory cytokines such as interferon‐gamma (IFN‐*γ*) and interleukin‐17A (IL‐17A).^[^ ^4^ ^]^ Therefore, preventing the migration of CD4^+^ T lymphocytes to the CNS is an important strategy for preventing MS progression.^[^ ^5^ ^]^

**Figure 1:**
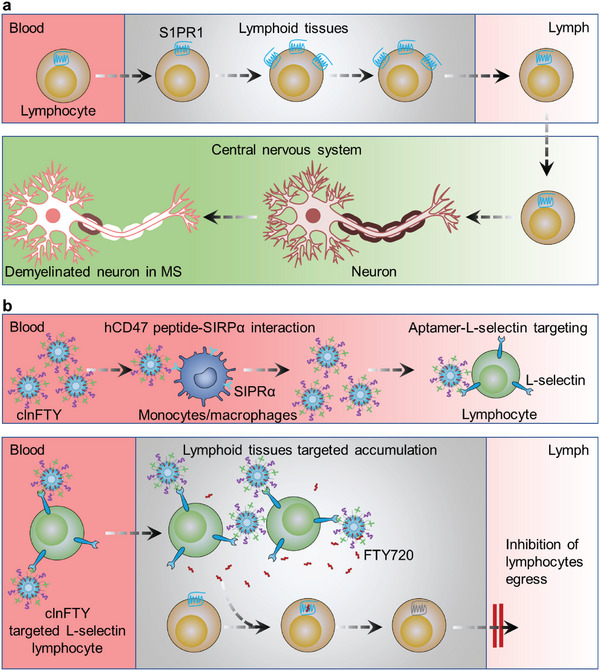
A schematic illustration of an L‐selectin expressing lymphocytes targeted nanosystem for delivering immunosuppressive drugs to LNs for MS treatments. a) During MS progression, S1PR1 is critical for T cells efflux from lymphoid tissues. These T cells infiltrate the central nervous system and cause demyelination of neuronal axons. b) clnFTY nanoparticles can interact with SIRP*α* on monocytes/macrophages via CD47 peptide, thereby preventing macrophage clearance and prolonging circulation in vivo. Meanwhile, clnFTY nanoparticles target lymphocytes expressing L‐selectin for targeted LN delivery of FTY720. Following delivery to the LNs, FTY720 promotes S1PR1 internalization in lymphocytes and inhibits the entry of pathogenic lymphocytes into the blood.

Today, different MS treatment options are available to influence and modulate the immune response through various mechanisms.^[^ ^6^ ^]^ Most of these treatments focus on lymphocytes, but cause some potential side effects and rapid recovery of lymphocytes after treatment stops.^[^ ^7^ ^]^ FTY720 (also known as fingolimod), a sphingosine‐1‐phosphate receptor (S1PR) modulator, is a disease modifying therapy (DMT) drug approved for the clinical remission of MS progression.^[^ ^8^ ^]^ FTY720 binds to the S1PR1 on the lymphocyte membrane and leads to its internalization and degradation, thereby blocking lymphocytes in the LNs (**Figure** **1**a).^[^ ^9^ ^]^ However, although S1PRs are most abundant in lymphocytes and lymphoid tissues, they are also widely expressed in various cell types in other organs, including the heart, brain, liver, and possibly the retina.^[^ ^10^ ^]^ Treatment with FTY720 may result in a wide range of adverse reactions including hypertension, heart block, bradycardia and macular edema.^[^ ^11^ ^]^ In addition, the need for daily dosing of FTY720 can lead to poor patient compliance. Expression of the S1PR on T cells can be restored after cessation of FTY720 treatment, exacerbating MS progression.^[^ ^12^ ^]^ Thus, effectively delivering these immunosuppressive drugs directly to LNs can improve their duration of action and therapeutic effect, and avoid potential side effects. Different entry routes for LN drug delivery have been explored, including active transport through targeting dendritic cells (DCs), passive transport through lymphatic vessels, and direct LNs injection.^[^ ^13^ ^]^

In this study, we designed a nanoplatform to target L‐selectin expressing lymphocytes for LN‐targeted delivery of FTY720. FTY720 is a synthetic structural analog of the cellular bioactive sphingosine, a sphingolipid found in plant and animal cell membranes. Inspired by this, liposomes were used to deliver FTY720 by self‐assembling FTY720 in lipid membrane (nFTY). For the targeted delivery of FTY720 in LNs, this study proposed a novel strategy to target L‐selectin expressing lymphocytes (Figure 1). L‐selectin has been reported to be a homing receptor of lymphocytes to peripheral LNs.^[^ ^14^ ^]^ Therefore, L‐selectin targeting aptamers were decorated on nFTY nanoparticles (lnFTY). In addition, to improve the chances of targeting L‐selectin‐expressing lymphocytes, CD47 peptides were decorated on the lnFTY nanoparticles (clnFTY). CD47 can interact with signal regulatory protein alpha (SIRP*α*) on the monocytes/macrophage membrane to constitute a “don't eat me” signal, blocking the uptake of clnFTY by monocytes/macrophages, and enhancing its circulation in vivo (Figure 1).^[^ ^15^ ^]^ Owing to the efficient delivery of FTY720 to LNs, clnFTY nanoparticles efficiently inhibited lymphocyte infiltration of the CNS and alleviated injury in the EAE mice, which is the most frequently used model in MS research. Especially, when combined with a ketogenic diet, clnFTY nanoparticles suppressed CNS damage in EAE mice for at least 42 days with just one treatment. Since FTY720 must be taken every day to inhibit the occurrence of EAE, clnFTY greatly increases the treatment cycle of FTY720 to exert its effect of blocking lymphocytes in LNs.

## 2 Results and Discussion

### 2.1 Preparation and Characterization of clnFTY Nanoparticles

**Figure 2:**
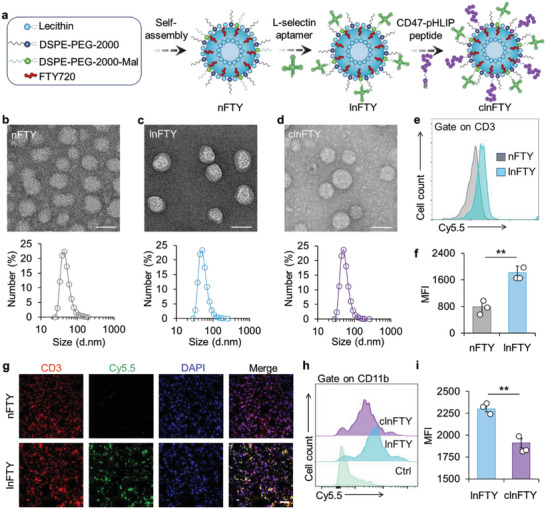
The characterization of clnFTY nanoparticles. a) A schematic illustration of the preparation of clnFTY nanoparticles. b) TEM images showing the size distribution of nFTY, c) lnFTY, and d) clnFTY nanoparticles. Scale bars, 50 nm. e) Flow cytometry data and f) quantitative analysis of T cells incubated with Cy5.5‐labeled nFTY and lnFTY nanoparticles. g) Confocal images of T cells treated with Cy5.5‐labeled nFTY or lnFTY nanoparticles. T cells were incubated at 4 °C to reduce their metabolic activity. Scale bar, 20 µm. h) Flow cytometry data and i) quantitative analysis of intracellular fluorescence of Cy5.5‐labeled lnFTY and clnFTY nanoparticles in macrophages. Data are presented as mean ± standard error of the mean (SEM, *n* = 3), and statistical significance was assessed by the unpaired two‐tailed Student's *t*‐test, **p < 0.01.

nFTY nanoparticles were prepared by assembling FTY720 with lecithin, DSPE‐PEG‐2000, and cholesterol (**Figure** **2**a). L‐selectin targeting aptamers were decorated on nFTY nanoparticles by click chemistry using DSPE‐PEG‐2000‐MAL lipid (Figure 2a). To decorate lnFTY nanoparticles with CD47 peptides, pH‐(Low) Insertion Peptides (pHLIPs) were attached to the end of CD47 peptides (CD47‐pHLIP). After incubating CD47‐pHLIP peptides and lnFTY nanoparticles in the pH 6.5 solution, the pHLIP moiety can be automatically inserted into the lnFTY nanoparticles (Figure 2a). The encapsulation of FTY720 in nFTY nanoparticles was determined by high‐performance liquid chromatography (HPLC). Interestingly, almost 100% of the FTY720 was encapsulated in nFTY nanoparticles (Figure S1, Supporting Information). Tetramethylrhodamine (TRITC) labeled aptamers were used to determine the ligation efficiency. As shown in Figure S2a, Supporting Information, the ligation efficiency of L‐selectin targeting aptamers is about 65% according to the standard curve. Likewise, the insertion efficiency of CD47‐pHLIP was determined by fluorescence, with an efficiency of about 90% (Figure S2b, Supporting Information). Next, the morphology and size distribution of nFTY, lnFTY, or clnFTY were investigated using transmission electron microscopy (TEM) and dynamic light scattering (DLS). As shown in Figure 2b–d, TEM images indicated that nFTY, lnFTY, and clnFTY nanoparticles exhibited well‐dispersed spherical morphologies. DLS data showed that the hydrodynamic size of nFTY nanoparticles is about 44 nm and increased to about 50 nm after decoration of L‐selectin targeting aptamer (Figure 2b–d). Further insertion of CD47‐pHLIP peptides did not obviously change the size of the nanoparticles (Figure 2b–d). The zeta potential of nFTY, lnFTY, and clnFTY nanoparticles was −23.27, −24.13, and −25.23 mV, respectively (Figure S3, Supporting Information).

L‐selectin is expressed in naive and central memory T cells for migration back to LNs.^[^ ^16^ ^]^ Next, the targeting capability of lnFTY nanoparticles was studied using CD3^+^ T cells isolated from LNs. Cyanine 5.5 (Cy5.5) was encapsulated in the hydrophilic core to aid in detection. As shown in Figure 2e,f, flow cytometry analysis showed that decorative L‐selectin targeting aptamers (lnFTY) significantly increased the uptake of nanoparticles in CD3^+^ T cells compared with the nFTY group. To further confirm this, CD3^+^ T cells were incubated at 4 °C to reduce their metabolic activity, thereby minimizing the cellular uptake of nanoparticles. However, lnFTY nanoparticles should be able to target and bind to L‐selectin on the membrane of T cells. As shown in Figure 2g, the fluorescence of Cy5.5 was barely detectable in nFTY‐treated CD3^+^ T cells but strongly increased in the lnFTY group, confirming L‐selectin's targeting ability of lnFTY nanoparticles. CD47 peptide decorated on nanoparticles can bind to SIRP*α* on monocytes/macrophages to inhibit the phagocytosis of nanoparticles, thereby promoting the circulation time of nanoparticles in vivo. In this study, a human CD47 peptide was used, which did not match mouse SIRP*α*.^[^ ^17^ ^]^ Therefore, CD11b^+^ macrophages were isolated from humanized SIRP*α* mice (HS mice) to study the phagocytosis of nanoparticles. Indeed, flow cytometry analysis revealed that the insertion of CD47‐pHLIP peptides into lnFTY nanoparticles significantly reduced the uptake of nanoparticles in CD11b^+^ macrophages (Figure 2h,i). Moreover, FTY720 can be released from nFTY, lnFTY, or clnFTY nanoparticles in a sustained manner (Figure S4, Supporting Information). Together, these data demonstrated that we have successfully synthesized FTY720 nanodrugs with L‐selectin targeting and self‐passivation capabilities.

### 2.2 Targeting L‐Selectin Expressing T Cells for LN‐Directed Delivery of FTY720 Nanoparticles

**Figure 3:**
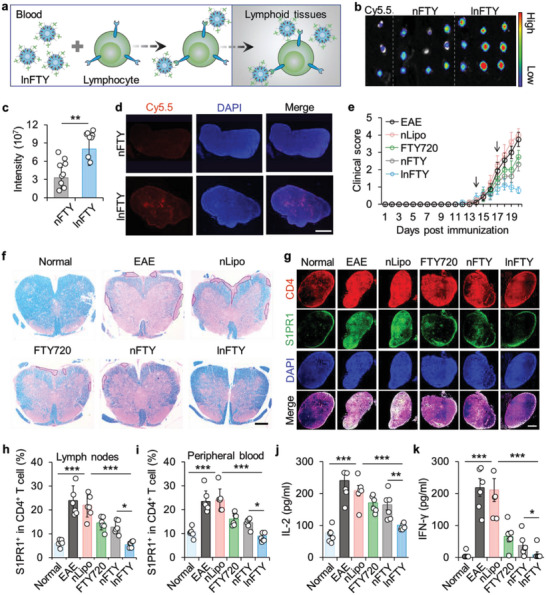
Targeting L‐selectin T cells for LN‐directed delivery of FTY720. a) A schematic illustration of targeting L‐selectin on lymphocytes for directed delivery of lnFTY nanoparticles. b) Ex vivo imaging and c) quantitative analysis of fluorescence in LNs from mice treated with free Cy5.5, Cy5.5‐labeled nFTY, or lnFTY for 24 h. d) Immunofluorescence images of LNs from mice treated with Cy5.5‐labeled nFTY or lnFTY for 24 h. Scale bar: 100 µm. e) Clinical scores of EAE mice treated with nLipo, free FTY720, nFTY, or lnFTY nanoparticles. Arrows indicate days of drug formulation treatment. f) LFB staining of spinal cords from mice with various treatments. Scale bar, 500 µm. g) Immunofluorescence images of S1PR1 and CD4^+^ T cells in LNs from mice with various treatments. Scale bar, 100 µm. h) Proportion of S1PR1^+^ in CD4^+^ T cells in LNs and i) peripheral blood from various formulation‐treated mice. j) The detection of the inflammatory cytokines IL‐2 and k) IFN‐*γ* in serum collected from mice receiving various treatments. Data are presented as mean ± standard error of the mean (S.E.M., *n* = 3 in c; *n* = 6 in e, h, i, j, and k). Statistical significance was assessed by unpaired two‐tailed Student's *t*‐test for c, or by ANOVA with Newman–Keuls test for h, i, j, and k. *p < 0.05, **p < 0.01, ***p < 0.001.

Having confirmed the L‐selectin targeting capability of lnFTY nanoparticles in vitro, we next examined whether L‐selectin expressing T cells could deliver lnFTY nanoparticles to LNs (**Figure** **3**a). We first investigated the proportions of L‐selectin^+^ T cells in peripheral blood and LNs in nFTY‐ and lnFTY nanoparticles‐treated mice. As shown in Figure S5, Supporting Information, the percentage of L‐selectin^+^CD3^+^ T cells in lnFTY group did not significantly change compared with the nFTY group, neither in peripheral blood nor in LNs, confirming that decoration of L‐selectin targeting aptamer should not affect the trafficking of L selectin‐positive T cells (Figure S5, Supporting Information). Then, Cy5.5‐labeled nFTY or lnFTY nanoparticles were injected intravenously into C57BL/6J mice, and inguinal LNs were isolated for ex vivo imaging. As shown in Figure 3b, IVIS imaging showed that the fluorescence intensity was minimal in free Cy5.5 treated mice and increased in nFTY treated mice 24 h after administration. Importantly, LNs isolated from lnFTY‐treated mice exhibited stronger fluorescence than the nFTY group, confirming that targeting L‐selectin‐expressing T cells enhanced the LN accumulation of nanoparticles (Figure 3b,c). The distribution of nFTY or lnFTY nanoparticles in LNs was examined using confocal microscopy, showing that more nanoparticles accumulated inside the LNs of lnFTY‐treated mice than in the nFTY‐treated mice (Figure 3d). Furthermore, confocal images showed that most of the lnFTY nanoparticles were distributed on T cell membrane, suggesting that T cells might not take up lnFTY nanoparticles (Figure S6, Supporting Information). Meanwhile, CD11c^+^DCs were also stained, showing that almost no lnFTY nanoparticles were distributed in DCs (Figure S6, Supporting Information). We further confirmed the LNs targeting ability using HPLC, showing the amount of FTY720 in lnFTY nanoparticles‐treated LNs was greater than other groups (Figure S7, Supporting Information).

Next, CD4^+^ T cells were isolated from the LNs and treated with empty liposomes (nLipo), free FTY720, nFTY, or lnFTY nanoparticles to examine the expression of S1PR1.^[^ ^18^ ^]^ As shown in Figure S8, Supporting Information, the expression of S1PR1 in the CD4^+^ T cells was significantly increased after stimulation with CD3 and CD28, and nLipo treatment did not affect the expression of S1PR1. FTY720 could downregulate the expression of S1PR1 in CD4^+^ T cells and was not affected after nFTY formation (Figure S8, Supporting Information). Importantly, lnFTY nanoparticles exhibited the lowest expression of S1PR1 compared to other groups, which could be due to the T cell targeting capability. Moreover, the activation of the AKT‐STAT3 pathway plays an important role in T cell mediated inflammation, which can also be regulated by FTY720. Western blotting results showed that FTY720 or nFTY720 nanoparticles downregulated the expression of phosphorylated AKT or STAT3 (p‐AKT or p‐STAT3, respectively) proteins, and this effect was further enhanced by treatment with lnFTY nanoparticles of stimulated CD4^+^ T cells (Figure S9, Supporting Information). Activation of the AKT‐STAT3 pathway also mediates the production of Th1 and Th17 cells associated cytokines such as IL‐2, IFN‐*γ*, and IL‐17A.^[^ ^19^ ^]^ Therefore, we first examined the gene expression of these inflammatory cytokines, showing that IL‐2, IFN‐*γ*, and IL‐17A related genes were upregulated after stimulation but downregulated with lnFTY treatment (Figure S10, Supporting Information). Accordingly, the secretion of IL‐2, IFN‐*γ*, and IL‐17A was increased in the stimulated group (Figure S10, Supporting Information). Although FTY720 or nFTY reduced the secretion of these inflammatory cytokines, the lowest levels of IL‐2, IFN‐*γ*, and IL‐17A were obtained in lnFTY nanoparticles treated T cells (Figure S10, Supporting Information).

The EAE model of MS was used to investigate the remission of MS progression.^[^ ^20^ ^]^ EAE mice were obtained by immunizing proteins derived from the myelin sheath (i.e., myelin oligodendrocyte glycoprotein, MOG) accompanied by an intraperitoneal injection of pertussis toxin (PTX) on day 2. Clinical scores were recorded daily, and ascending paralysis occurred on day 14 after immunization (Figure 3e). Then, EAE mice were randomly divided into five groups: EAE only, nLipo, FTY720, nFTY, and lnFTY groups. The frequency of the clinical use of FTY720 is once a day, which can inhibit disease progression, but long‐term use may cause serious organ damage to MS patients.^[^ ^21^ ^]^ In this study, to assess the importance of LN‐targeted FTY720 delivery, a twice‐weekly dosing regimen was used on days 14 and 17. As the disease continued to advance, the clinical signs of the vehicle‐treated EAE group peaked at days 17–20 and nearly 40% of the mice died (Figure S11, Supporting Information). Treatment with FTY720 or Lipo‐F temporarily inhibited the progression of EAE, but the clinical score increased 1 day after drug withdrawal (Figure 3e). In contrast, lnFTY treatment had a sustained therapeutic effect, with significant reductions in clinical scores and lethality (Figure 3e). These results indicate that targeted delivery to LNs is a useful strategy to enhance the efficacy of FTY720 treatment.

Luxol fast blue (LFB) staining was used to assess the severity of demyelination of the spinal cord white matter.^[^ ^22^ ^]^ As shown in Figure 3f, LFB staining of a normal mouse spinal cord showed uniform white matter. In contrast, perivascular focal areas without LFB staining were more frequently observed in EAE mice, suggesting the successful construction of MS model (Figure 3f). Treatment with nLipo had minimal effects on myelin loss in EAE mice (Figure 3f). Free FTY720 or nFTY treatment slightly increased the LFB staining of white matter, and lnFTY exhibited the best therapeutic results compared with the other groups (Figure 3f). Furthermore, LNs from all groups were examined for CD4^+^ T cell phenotypes, which are considered the main pathogenic immune cells in MS.^[^ ^4^, ^23^ ^]^ As shown in Figure 3g, immunofluorescence staining showed that S1PR1‐positive T cells were obviously increased in the LNs of EAE mice compared with normal mice, and were unaffected by nLipo treatment. FTY720 or nFTY treatment slightly reduced the fluorescence intensity, while the lnFTY group had the lowest fluorescence signal (Figure 3g). Consistent with the immunofluorescence results, an increased proportion of S1PR1^+^CD4^+^ T cells in the LNs of EAE mice was detected by flow cytometry, indicating an inflammatory response (Figure 3h). Similarly, the percentage of S1PR1^+^CD4^+^ T cells was not affected by nLipo treatment, but was significantly reduced after FTY720 or nFTY treatment. Overall, lnFTY treatment was more effective than other groups in reducing S1PR1^+^CD4^+^ T cells in the LNs of EAE mice (Figure 3h). In addition, the expression of the transcription factor T‐bet in Th1 cells, which regulates cytokine IL‐2 and IFN‐*γ* expressions in Th1 cells,^[^ ^24^ ^]^ was elevated in the LNs of EAE mice and downregulated by FTY720 or nFTY nanoparticles treatment, but not as efficient as lnFTY (Figure S12, Supporting Information). Foxp3,^[^ ^25^ ^]^ a Treg cell biomarker, was expressed at a low level in all group (Figure S12, Supporting Information). These results demonstrate the immunopressive effect of lnFTY on effector T cells in the LNs of EAE mice. Inhibition of CD4^+^ T cell function in LNs strongly influenced the circulation of CD4^+^ T cells in the blood. We next investigated the percentage of CD4^+^ T cells in the peripheral blood of mice receiving various treatments. Indeed, circulating CD4^+^ T cells or S1PR1^+^CD4^+^ T cells in the peripheral blood were significantly increased in EAE mice compared with normal mice and were unaffected by nLipo treatment (Figure S13a, Supporting Information and Figure 3i). Treatment with FTY720 or nFTY reduced circulating S1PR1^+^CD4^+^ T cells in the peripheral blood, but not as effectively as lnFTY (Figure S13a, Supporting Information and Figure 3i). This suppression was further verified by examining secreted inflammatory cytokines, including IL‐2, IFN‐*γ*, and IL‐17A. As shown in Figure 3j,k and Figure S13b, Supporting Information, lnFTY treatment significantly reduced these inflammatory cytokine levels in the serum compared with other groups, confirming that lnFTY efficiently inhibited the inflammatory response induced by T cells. Major organs of all groups were also collected for histological analysis. As shown in Figure S14, Supporting Information, hematoxylin and eosin (H&E) staining showed that no obvious damage was observed in mice receiving the various treatments, which may be because FTY720 was only injected twice throughout the treatment period.

### 2.3 Escaping Macrophage Clearance Augments LN‐Directed Delivery of FTY720 Nanoparticles

**Figure 4:**
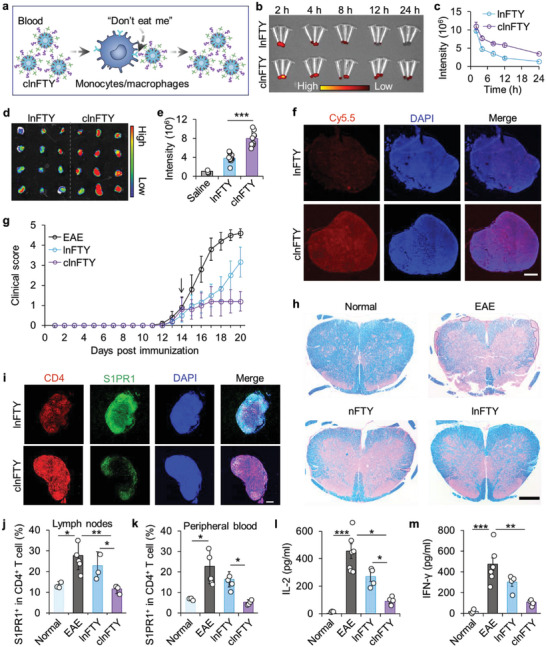
Escaping macrophage‐clearance augments LN‐directed delivery of FTY720. a) A schematic presentation of the self‐preservation capability of clnFTY nanoparticles. b) Ex vivo imaging and c) fluorescence intensity of Cy5.5 in blood from HS mice treated with Cy5.5‐labeled lnFTY or clnFTY nanoparticles. d) Ex vivo imaging and e) quantitative analysis of fluorescence in LNs from HS mice treated with Cy5.5‐labeled lnFTY or clnFTY for 48 h. f) Immunofluorescence images of LNs from HS mice treated with Cy5.5‐labeled lnFTY or clnFTY nanoparticles for 48 h. Scale bar, 100 µm. g) Clinical scores of EAE HS mice treated with lnFTY or clnFTY nanoparticles. Arrow indicates the day of drug formulation treatment. h) LFB staining of spinal cords collected from HS mice receiving various treatments. Scale bar, 500 µm. i) Immunofluorescence images of S1PR1 and CD4‐positive T cells in LNs from HS mice with various treatments. Scale bar, 100 µm. j) The proportion of S1PR1^+^ in CD4^+^ T cells in LNs and k) the peripheral blood from various formulation‐treated mice. l) Detection of inflammatory cytokines IL‐2 and m) IFN‐*γ* in serum collected from HS mice receiving various treatments. Data are presented as mean ± standard error of the mean (S.E.M., *n* = 3 in c; *n* = 3 for saline, *n* = 9 for lnFTY and clnFTY in e; *n* = 3 for normal, *n* = 5 for EAE, *n* = 4 for lnFTY and clnFTY in g, j, k, l, and m). Statistical significance was assessed by the unpaired two‐tailed Student's *t*‐test for e or by ANOVA with Newman–Keuls test for j, k, l, and m. *p < 0.05, **p < 0.01, ***p < 0.001.

In this study, FTY720‐loaded nanoparticles were designed to target L‐selectin expressing lymphocytes in peripheral blood for LN‐directed delivery. Therefore, maintaining nanoparticle levels in the blood long enough for them to reach circulating lymphocytes is crucial for LN targeting. As the primary scavenger cells of the body, monocytes/macrophages have the unique ability to phagocytose foreign particles to maintain cellular homeostasis as well as immune surveillance within the innate immune system.^[^ ^26^ ^]^ The CD47 peptide is thus used to constitute a “don't eat me” signal that blocks monocytes/macrophages uptake of clnFTY nanoparticles and enhances their circulation in vivo (**Figure** **4**a). To confirm this, Cy5.5‐labeled lnFTY and clnFTY nanoparticles were injected intravenously into HS mice, and blood was collected to examine circulation time. As shown in Figure 4b,c, IVIS images and quantitative analysis indicated that the cycle time of clnFTY nanoparticles was significantly longer than that of lnFTY nanoparticles, confirming the importance of self‐passivation. Next, LNs were collected from mice for ex vivo imaging. The fluorescence intensity of Cy5.5 in LNs from the clnFTY group was significantly stronger than that of the lnFTY group (Figure 4d,e). LNs were also sectioned for confocal imaging, further confirming that more clnFTY nanoparticles were accumulated in the LNs from the clnFTY group (Figure 4f).

To investigate the therapeutic effect of clnFTY nanoparticles, we induced an EAE model using HS mice and treated them with lnFTY and clnFTY nanoparticles only once on day 14. As shown in Figure 4g, lnFTY treatment attenuated the development of post‐treatment EAE, but clinical scores increased rapidly by the end of the experiment. Survival data also showed that lnFTY‐treated mice began to die on day 18 (Figure S15, Supporting Information). Importantly, clnFTY treated mice had stable clinical scores and no mice died after a single treatment (Figure 4g and Figure S10, Supporting Information). LFB staining of the spinal cords showed that clnFTY treatment preserved white matter integrity better than lnFTY treatment (Figure 4h). Immunofluorescence staining of S1PR1 in the LNs confirmed that clnFTY nanoparticles strongly downregulated the expression of S1PR1 in LNs compared with lnFTY treatment (Figure 4i). Confocal images showed that T‐bet expression in clnFTY‐treated LNs was decreased compared with lnFTY group and Foxp3 expression was not affected (Figure S16, Supporting Information). This is unsurprising since clnFTY nanoparticles can accumulate in LNs more efficiently than lnFTY nanoparticles (Figure 4d). Flow cytometry analysis also indicated that clnFTY treatment significantly decreased the percentage of S1PR1^+^CD4^+^ T cells in LNs compared with the lnFTY group (Figure 4j). As a result, CD4^+^ T cells or S1PR1^+^CD4^+^ T cells in peripheral blood were significantly reduced in the clnFTY group, which could be due to the blockade of lymphocytes in the LNs (Figure 4k and Figure S17a, Supporting Information). Detection of inflammatory cytokines also showed that clnFTY treatment significantly reduced the secretion of IL‐2, IFN‐*γ*, and IL‐17A in the serum of EAE HS mice (Figure 4l‐m and Figure S17b, Supporting Information). H&E staining of major organs from mice treated with lnFTY and clnFTY nanoparticles showed no obvious damages, suggesting the excellent biocompatibility of lnFTY and clnFTY nanoparticles (Figure S18, Supporting Information).

### 2.4 Ketogenic Diet and clnFTY Treatments Durably Suppression of EAE Progression

**Figure 5:**
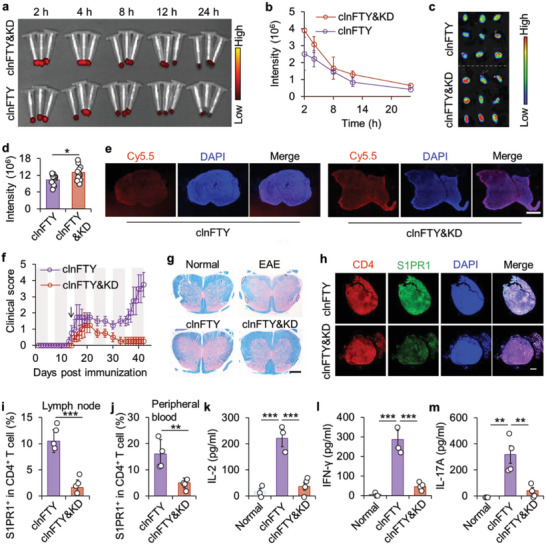
A ketogenic diet and clnFTY treatments extended the suppression of EAE progression. a) Ex vivo images and b) calculation of the levels of clnFTY nanoparticles in blood collected from HS mice treated without or with a ketogenic diet (clnFTY&KD) after injection of Cy5.5‐labeled clnFTY nanoparticles. c) Ex vivo imaging and d) quantitative analysis of fluorescence in LNs from HS mice from the clnFTY&KD groups. e) Immunofluorescence staining of LNs from HS mice of the clnFTY and clnFTY&KD groups. Scale bar, 100 µm. f) Clinical scores of EAE HS mice treated with clnFTY or clnFTY&KD. The shaded part indicates the days with ketogenic diet treatments, and the arrow indicates the day of drug formulation treatment. g) LFB staining of spinal cords collected from HS mice with various treatments. Scale bar, 500 µm. h) Immunofluorescence staining of S1PR1 and CD4‐positive T cells in LNs from HS mice with clnFTY and clnFTY&KD treatments. Scale bar, 100 µm. i) Proportion of S1PR1^+^ in CD4^+^ T cells in LNs or j) the peripheral blood of HS mice with clnFTY and clnFTY&KD treatments. k) Detection of inflammatory cytokines IL‐2, l) IFN‐*γ*, and m) IL‐17A in the serum of mice with clnFTY and clnFTY&KD treatments. Data are presented as mean ± standard error of the mean (S.E.M., *n* = 3 in b; *n* = 9 in d; *n* = 4 for f, i, j, k, l, and m). Statistical significance was assessed by the unpaired two‐tailed Student's *t*‐test for d, i, and j or by ANOVA with Newman–Keuls test for k, l, and m. *p < 0.05, **p < 0.01, ***p < 0.001.

A low‐carbohydrate high‐fat ketogenic diet has become an intervention for many neurological diseases, such as epilepsy, Alzheimer's disease (AD), Parkinson's disease (PD), sleep disorders, brain cancer, and amyotrophic lateral sclerosis (ALS).^[^ ^27^ ^]^ In addition, ketogenic diets have been reported to modulate immune system responses.^[^ ^28^ ^]^ Therefore, a ketogenic diet was investigated regarding its effect on clnFTY nanoparticles for treating EAE HS mice. First, we examined the circulation of clnFTY nanoparticles in HS mice fed with a normal or ketogenic diet. After 2 weeks of an intermittent ketogenic diet, HS mice were injected with Cy5.5‐labeled clnFTY nanoparticles. As shown in **Figure** **5**a, the circulation of clnFTY nanoparticles in the ketogenic diet‐fed HS mice (clnFTY&KD) was similar to that in the normal diet group, indicating that a ketogenic diet did not affect the in vivo circulation of clnFTY nanoparticles. Interestingly, ex vivo imaging of LNs showed that the fluorescence intensity of Cy5.5 in LNs from clnFTY&KD was significantly increased compared with the clnFTY group (Figure 5c,d). This is probably due to the enhanced uptake of clnFTY nanoparticles in L‐selectin T lymphocytes. As shown in Figure S19, Supporting Information, the cellular uptake of clnFTY nanoparticles in T cells was significantly augmented by culturing in a ketogenic diet mimic medium (containing *β*‐hydroxybutyric acid, the main metabolite of a ketogenic diet).^[^ ^29^ ^]^ Similarly, confocal imaging of LNs showed stronger fluorescence of Cy5.5 in LNs from the clnFTY&KD group (Figure 5e).

Next, EAE HS mice were obtained and the clnFTY&KD group was given an intermittent ketogenic diet 2 weeks in advance. clnFTY nanoparticles were administered only once on day 14. As shown in Figure 5f, the clinical scores of the clnFTY group quickly increased after day 30. In contrast, the clinical scores of the clnFTY&KD group continued to decrease after injection of clnFTY nanoparticles and almost completely recovered after day 35 (Figure 5f). During the 42 days of observation, the clinical scores of the clnFTY&KD group did not increase, and no mice died, while half of the mice in the clnFTY group died on day 42 (Figure 5f and Figure S20, Supporting Information). LFB staining of spinal cords also showed some demyelination of spinal cords in the clnFTY group, and LFB staining of the spinal cord in the clnFTY&KD group was relatively normal (Figure 5f). Immunofluorescence results also showed downregulated expression of S1PR1 in the LNs from the clnFTY&KD group, revealing that the function of FTY720 was still maintained at day 42 (Figure 5h). Confocal images showed that T‐bet expression in clnFTY&KD ‐treated LNs was decreased compared with clnFTY group and Foxp3 expression was not affected (Figure S21, Supporting Information). This is further confirmed by flow cytometry, showing significantly fewer S1PR1^+^CD4^+^ T cells in the LNs from the clnFTY&KD group than in the clnFTY group (Figure 5i). As a result, circulating CD4^+^ T cells and S1PR1^+^CD4^+^ T cells in peripheral blood were significantly reduced in the clnFTY&KD group (Figure 5j and Figure S22, Supporting Information). Inflammatory cytokine levels in the serum, including IL‐2, IFN‐*γ*, and IL‐17A, were also lower in the clnFTY&KD treated EAE HS mice, suggesting that inflammation was inhibited in EAE HS mice (Figure 5k,m). Last, major organs of various groups were collected for H&E, suggesting that clnFTY treatment and a ketogenic diet caused no obvious damage (Figure S23, Supporting Information). Taken together, these results indicate that clnFTY nanoparticles, in combination with a ketogenic diet exhibit superior capability to durably suppression of the progression of EAE.

### 2.5 Therapeutic Study of clnFTY Nanoparticles and Ketogenic Diet on Immune Cells from MS Patients

**Figure 6:**
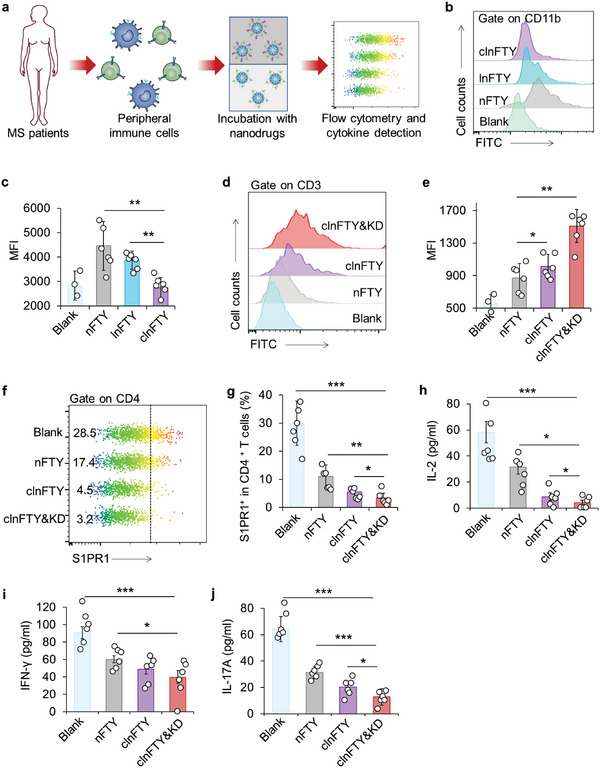
Therapeutic study of the effect of clnFTY nanoparticles and a ketogenic diet on the immune cells of MS patients. a) PBMCs isolated from MS patients in the relapsing phase were used to examine the therapeutic ability of clnFTY nanoparticles. b) Flow cytometry data and c) quantitative analysis of the intracellular nFTY, lnFTY, or clnFTY nanoparticles in macrophages from MS patients. d) Flow cytometry data and e) quantitative analysis of the intracellular fluorescence in T cells from MS patients with various treatments. f) Flow cytometry data and g) quantitative analysis of S1PR1^+^ in CD4^+^ T cells from MS patients with various treatments. h) Detection of inflammatory cytokines IL‐2, i) IFN‐*γ*, and j) IL‐17A in supernatant PBMCs with various treatments. Data are presented as mean ± standard error of the mean (S.E.M., *n* = 6). Statistical significance was assessed by ANOVA with Newman–Keuls test. *p < 0.05, **p < 0.01, ***p < 0.001.

To further verify the therapeutic ability of clnFTY nanoparticles, peripheral blood mononuclear cells (PBMCs) were collected from MS patients in the relapsing phase and treated with various nanoparticles (**Figure** **6**a). First, macrophages were isolated to examine the self‐passivation capability of clnFTY nanoparticles. The cellular uptake of fluorescein isothiocyanate‐labeled (FITC) nFTY, lnFTY, or clnFTY nanoparticles in macrophages was determined by flow cytometry. As shown in Figure 6b,c, the fluorescence intensity in macrophages increased after incubation with nFTY nanoparticles and did not significantly change after aptamer modification. Amazingly, clnFTY nanoparticles showed the lowest fluorescence intensity, suggesting that CD47 peptide modification can help nanoparticles evade clearance by macrophages (Figure 6b,c). Next, we confirmed the T cell targeting ability of clnFTY nanoparticles using patient PBMCs. According to the flow cytometry data, the fluorescence intensity in T cells with clnFTY nanoparticles treatment was significantly stronger than that of the nFTY group, probably because clnFTY nanoparticles could target the L‐selectin expressing T cells (Figure 6d,e). Interestingly, this targeting ability was significantly enhanced if PBMCs were cultured in the ketogenic diet mimic medium, consistent with previous results (Figure 6d,e). These data suggest that the design in this study to self‐inactivate or target L‐selectin‐expressing T cells may be feasible in MS patients.

Last, S1PR1 expression in CD4^+^ T cells was examined after nFTY, clnFTY, or clnFTY&KD treatment. As shown in Figure 6f,g, the percentage of S1PR1^+^CD4^+^ T cells was about 30%, which decreased to about 15% after nFTY treatment. Only about 5% of CD4^+^ T cells were positive for S1PR1 in the clnFTY group, confirming the importance of T cells targeting (Figure 6f,g). Overall, the clnFTY&KD group exhibited the lowest number of S1PR1^+^CD4^+^ T cells (Figure 6f,g). Detection of inflammatory cytokines confirmed that PBMCs from MS patients could secrete IL‐2, IFN‐*γ*, and IL‐17A, indicating that these immune cells were activated and in an inflammatory state (Figure 6h–j). Treatment with clnFTY nanoparticles showed better inhibitory efficiency than nFTY treatment, and these cytokines were greatly decreased in the clnFTY&KD group. Taken together, these results reveal that clnFTY nanoparticles can effectively regulate inflammatory T cells, especially under ketogenic diet condition.

## 3 Conclusion

In this study, we developed a nanoplatform for delivering immunosuppressive drugs to LNs for durable MS treatment. Targeted LN delivery was achieved by targeting lymphocytes expressing L‐selectin, a homing receptor for T cells entering LNs. Therefore, L‐selectin targeting aptamer was modified on the nanoparticles encapsulated with FTY720. Lymphocytes then delivered the nanoparticles to the LNs by targeting L‐selectin. To minimize macrophage‐phagocytosis of nanoparticles to increase circulation time, human CD47 peptide was also modified on the nanoparticles to create a “don't eat me” signal. Together, clnFTY nanoparticles could efficiently deliver FTY720 to LNs and delay disease progression in EAE HS mice after a single dose of clnFTY treatment. To further improve the therapeutic efficiency, a low‐carbohydrate high‐fat ketogenic diet, was used to enhance the LN targeting and curative efficacy of clnFTY nanoparticles. Importantly, there was no increase in clinical scores in the clnFTY&KD treated EAE HS mice during the 42‐day observation period, which was obtained after only a single dose. By encapsulating FTY720 in clnFTY nanoparticles, the therapeutic efficacy of FTY720 was greatly enhanced compared to daily dosing of free drugs, especially when combined with a ketogenic diet. Last, clinical samples were collected and the targeting ability of clnFTY nanoparticles to T cells, as well as S1PR1 expression and inflammatory T cell regulation ability were confirmed. This study provides insight into the rational delivery of immunosuppressive drugs for durable MS treatment.

## 4 Experimental Section

### Materials

For cell culture and stimulation, RPMI 1640 medium, penicillin/streptomycin, fetal bovine serum (FBS), phosphate buffered saline (PBS), and sulfoxide (DMSO) were purchased from Beyotime (Shanghai, China). Anti‐mouse CD3*ε* and CD28 antibody (Biolegend, 100 339, 102 115) were purchased from Kasma Mall (www.casmart.com.cn).

For cellular and molecular biological studies, SDS‐PAGE gel, polyvinylidene difluoride (PVDF), TBST, defatted milk powder, 4% paraformaldehyde, polyethylene glycol 2000 (PEG2000), Triton X‐100 were purchased from Solarbio Science & Technology Co., Ltd (Beijing, China). Percoll, Cell Counting Kit‐8 (CCK‐8), RIPA lysates, BCA Protein Quantitation Kit, ECL western blotting substrate and goat serum were purchased from Beyotime Biotechnology Co., Ltd (Shanghai, China). The TRIzol reagent (Invitrogen, 15596‐026), SuperScript VILO cDNA Synthesis Kit (ThermoFisher, 11 754 250), ELISA kit mouse IL‐2 (MEIMIAN, MM‐0701 M), mouse IFN‐*γ* (MEIMIAN, MM‐45169 M), mouse IL‐17A (MEIMIAN, MM‐0759 M), human IL‐2 (MEIMIAN, MM‐0055H), human IFN‐*γ* (MEIMIAN, MM‐0033H), and human IL‐17A (MEIMIAN, MM‐2117H) were purchased from Kasma Mall.

Antibodies used in this study: p‐AKT Ser473 (Proteintech, 66444‐1‐Ig); AKT (Proteintech, 60203‐2‐Ig); p‐STAT3 Tyr705 (CST, 9145); STAT3 (CST, 12 640); S1PR1 (Abcam, ab11424); T‐bet (Proteintech, 13 700); Foxp3 (Proteintech, 65 089); mouse APC‐CD3 (Biolegend, 100 236); mouse PE‐CD4 (Biolegend, 100 408); human APC‐CD3 (Biolegend, 317 317); human PE‐CD4 (Biolegend, 980 804); Brilliant Violet 605‐CD11b (Biolegend, 101 237); PE‐CD11c (Biolegend, 117 038); APC‐CD62L(Biolegend, 104 411); GAPDH (Proteintech, 10494‐1‐AP); horseradish peroxidase (HRP)‐conjugated rabbit secondary antibody (Solarbio, SE134); HRP‐conjugated mouse secondary antibody (Solarbio, K0055G‐HRP); Alexa Fluor 488 conjugated anti‐rabbit IgG (CST, 4408), and Alexa Fluor 488 conjugated anti‐mouse IgG (CST, 4410).

Mice: C57BL/6J mice were purchased from SPF Biotechnology (Beijing, China). Humanized SIRP*α* (HS) mice were purchased from GemPharmatech LLC (Nanjing, China). Ketogenic diet were obtained from Trophic Animal Feed High‐Tech Co.,Ltd (Haian, China). MOG_35–55_ (Scilight‐peptide, SL2134‐2), complete Freund's adjuvant (Sigma‐Aldrich, F5881), Mycobacterium tuberculosis (BD Pharmingen, 231 141) and pertussis toxin (Listlabs, PTX‐181) were purchased from Kasma Mall. All animal experiments were performed in accordance with the relevant rules and regulations, and protocol was approved by Institutional Animal Care and Use Committee of National Center for Nanoscience and Technology (NCNST21‐2103‐0403).

### Clinical Sample Collection

Sample collection was approved by the Third Affiliated Hospital of Sun Yat‐sen University ([2022]02‐490). Patients with established diagnosis of MS were included. All participants signed written informed consent. Blood from MS patients were collected and diluted with PBS. PBMCs were enriched based on 70% Percoll gradients and centrifugation at 800 g for 30 min.

### Preparation of Nanoparticles

For nFTY nanoparticles, lecithin, cholesterol, and DSPE‐PEG‐2000 were dissolved in dichloromethane at a mass ratio of 30:5:4. The solution was evaporated in a round bottom flask under reduced pressure using a vacuum rotary evaporator to from a thin film. Nanoparticles were obtained by hydration with PBS solution and sonication. For lnFTY nanoparticles, DSPE‐PEG‐2000 was replaced by a mixture of DSPE‐PEG‐2000 and DSPE‐PEG‐2000‐Mal (5:1) in the first step. Then, nanoparticles were reacted with L‐selectin targeting aptamer (5′‐SH‐C6‐TAGCCAAGGTAACCAGTACAAGGTGCTAAACGTAATGGCTTCGGCTTAC‐3′) overnight at room temperature. For clnFTY nanoparticles, the pH of lnFTY nanoparticles solution was adjusted to 6.5, and clnFTY nanoparticles were obtained by mixed lnFTY nanoparticles with CD47‐pHLIP peptides (sequence of CD47 targeting peptide:

GNYTCEVTELTREGETIIELK; sequence of pHLIP: AEQNPIYWARYADWLFTTPLLLLDLALLVDADEGT; sequence of CD47‐pHLIP: GNYTCEVTELTREGETIIELK‐PEG4‐AEQNPIYWARYADWLFTTPLLLLDLALLVDADEGT) for 0.5 h at room temperature. To remove free aptamers or CD47‐pHLIP peptides, nanoparticles were purified using 10 KD ultrafiltration tubes (4000 g, 20 min) and washed with PBS solution for at least twice.

### Characterization of Nanoparticles

The hydrodynamic sizes of nFTY, lnFTY, and clnFTY nanoparticles (2 mg mL^−1^) were measured by ZetaSizer Nano series Nano‐ZS (Malvern Instruments, Malvern, UK). The morphology of nanoparticles was characterized using an HT‐7700 transmission electron microscope (Hitachi, Tokyo, Japan) after stained with 1% uranyl acetate for 7 min on the copper 200‐mesh grids. The encapsulation efficiency and loading capacity were determined with HPLC (Shimadzu, Kyoto, Japan).

### In Vivo Circulation and Ex Vivo LNs Imaging

Cy5.5‐labeled nFTY, lnFTY, and clnFTY nanoparticles (5 mg kg^−1^) were intravenously administered to healthy C57BL/6J or HS mice. Blood from mice was collected at 2, 4, 8, 12, and 24 h and imaged using IVIS Spectrum In Vivo Imaging System. Then, mice were sacrificed at 24 h in Figure 3 and 48 h in Figures 4 and 5. LNs were isolated for ex vivo imaging.

### Induction of EAE Model

C57BL/6J or HS mice at 6–8 weeks of age were used for the induction of EAE. The intermittent ketogenic diet was added 2 weeks in advance. The antigen emulsion was prepared by mixing an emulsion of MOG_35–55_ in PBS (50% w/v) with an equal volume of complete Freund's adjuvant, supplemented with 5 mg mL^−1^ Mycobacterium tuberculosis. Each C57BL/6J mouse was immunized by subcutaneous injection of 0.2 mL of antigen emulsion into its back, followed by an adjuvant injection of 2 µg of pertussis toxin in 0.1 mL on the dorsum of the foot. Due to difficulty in inducing EAE in SIRP*α*‐deficient mice, the dose was doubled in HS mice.^[^ ^30^ ^]^ The second dose of pertussis toxin was administered 2 days after the first immunization. Clinical signs of EAE mice were monitored daily. After the onset of disease (day 14), mice were injected intravenously with nLipo, free FTY720 (1 mg kg^−1^), nFTY (1 mg kg^−1^ of FTY720), lnFTY (1 mg kg^−1^ of FTY720), or clnFTY nanoparticles (1 mg kg^−1^ of FTY720) as indicated. During the experiments, mice were observed in a double‐blind manner every day and clinical scores were given according to the following criteria: 0, no clinical signs; 0.5, partial loss of tail tone; 1, affected tail tonus; 2, paresis of hind legs; 3, complete paralysis of the hind legs; 4, complete hind leg paralysis and foreleg paresis; and 5, death due to EAE. These criteria were established and modified according to previous clinical scale systems.^[^ ^31^ ^]^

### HE and LFB Staining

After the experiments, anesthetized mice were perfused intracardially with PBS followed by 4% paraformaldehyde. The mice were then scarified and the lumbar spinal cords were removed and fixed by immersion in 4% paraformaldehyde for 48 h. Cross‐sections (4 µm) taken from embedded spinal cord blocks were deparaffinized and stained with HE or LFB.

### Western Blot

The isolated CD4^+^ T cells were lysed on ice by RIPA lysates. Protein concentrations were determined with a BCA Protein Quantitation Kit. A total of 10–30 µg protein was loaded in each lane and was separated by 10% SDS‐PAGE. After separation, the proteins were transferred to PVDF membranes. The membranes were blocked with 5% defatted milk powder for 1 h. Blots were incubated overnight with the primary antibodies as follows: p‐AKT Ser473 (1:1000); AKT (1:1000); p‐STAT3 Tyr705 (1:500), and STAT3 (1:500). GAPDH (1:2000) was used as a control. Then horseradish peroxidase (HRP)‐conjugated secondary antibody was incubated with corresponding primary antibody blots for 1 h. Blots were visualized using the ECL western blotting substrate and recorded by the gel imager (Bio‐Rad, Hercules, USA).

### Immunohistochemistry

For immunohistochemistry staining, tissue sections were fixed with 4% paraformaldehyde for 15 min, and triton X‐100 (1%) were incubated with sections for 15 min to help the antibody permeating the cell membrane. Then, sections were blocked with 1% of goat serum for 1 h and incubated with primary antibodies at 4 °C overnight. After washed with PBS for three times, sections were incubated with Alexa Fluor 488 conjugated anti‐rabbit IgG and (1:2000) for 1 h. Next, DAPI (1:1000) was added for nuclei staining. The images were collected by confocal microscopy (Zeiss, Oberkochen, Germany) and analyzed by Image J software. Primary antibodies were used as following: S1PR1 (1:200); PE‐CD4 (1:200).

### Quantitative PCR

Total RNA was isolated using TRIzol reagent according to the manufacture's protocols. Then, cDNA was synthesized using a SuperScript VILO cDNA Synthesis Kit. Quantitative PCR was performed using the StepOnePlus Real‐Time PCR System (Life Technologies, New York, USA). The levels of gene expression were normalized to GAPDH. The specific primers (synthesized by GENEWIZ, Suzhou, China) were used as following:

*gapdh* (forward: TCTCTGCTCCTCCCTGTTCC; reverse: TACGGCCAAATCCG TTCACA);

*Il2* (forward: ATGAACTTGGACCTCTGCGG; reverse: GTCCACCACAGTTGCTG ACT);

*IFNG* (forward: AGCGCCAAGCATTCAATGAG; reverse: AATCTCTTCCCCACCC CGAA);

*Il17a* (forward: GCTGACCCCTAAGAAACCCC; reverse: GAAGCAGTTTGGGAC CCCTT).

### ELISA

Cell culture medium was centrifuged at 1500 rpm for 10 min and the supernatant was collected for examinations. For in vivo studies, the blood was centrifuged at 5000 rpm for 10 min and the supernatants were collected. The levels of IL‐2, IFN‐*γ*, and IL‐17A in each sample were measured by the scanning microplate reader (BioTek, Winooski, USA) using the Bradford method according to the manufacturer's instructions.

### Flow Cytometry

Lymphocyte suspensions from mouse blood were prepared with ACK lysis buffer or harvested from culture plates. After washing with PBS for three times, cell suspension was incubated with primary antibodies conjugated fluorescence and S1PR1 for 1 h. S1PR1‐labeled cells were then visualized using a fluorescent secondary antibody. Cells were analyzed on a FACS Aria III flow cytometer (BD Bioscience, Breda, The Netherlands). The following antibodies were used PE‐CD4 (0.5 µg mL^−1^), S1PR1 (1 µg mL^−1^), Alexa Fluor 488 anti‐rabbit (1 µg mL^−1^).

### Statistics and Reproducibility

All statistical data analyses were performed with Microsoft Excel or Graphpad Prism 6. All error bars used in this study are means ± S.E.M of at the least three independent experiments. For normally distributed data sets with equal variances, Student's *t*‐test was used for statistical analysis in the experiments with two groups, and ANOVA followed by Newman–Keuls test was used for statistical analysis in the experiments more than two groups. In all cases, significance was defined as *p* < 0.05.

## Conflict of Interest

The authors declare no conflict of interest.

## Author Contributions

Y.Z. and J.Z. contributed equally to this work. H.W. conceived the project; H.W., W.Q., and G.N. analyzed the data and wrote the manuscript; Y.Z. and J.Z. conducted all the experiments with help from X.C., W.H., S.S., and S.W. Y.H. went through clinical ethical procedures and collected clinical samples. All authors approved the manuscript.

## Supporting information

Supporting Information

## Data Availability

The data that support the findings of this study are available from the corresponding author upon reasonable request.
