# Supplementary material for: Targeting L‐Selectin Lymphocytes to Deliver Immunosuppressive Drug in Lymph Nodes for Durable Multiple Sclerosis Treatment
Source: Adv Sci (Weinh). 2023 May 12;10(20):2300738. doi: 10.1002/advs.202300738 (PMC10369270; doi:10.1002/advs.202300738)

ADVANCED SCIENCE
Open Access

Supporting Information

for *Adv. Sci.*, DOI 10.1002/advs.202300738

Targeting L-Selectin Lymphocytes to Deliver Immunosuppressive Drug in Lymph Nodes for Durable Multiple Sclerosis Treatment

Yipeng Zhao, Jie Zhang, Xi Cheng, Wenping Huang, Shishi Shen, Shilin Wu, Yiying Huang, Guangjun Nie, Hai Wang* and Wei Qiu**

Supporting Information

Targeting L-selectin Lymphocytes to Deliver Immunosuppressive Drug in Lymph Nodes for Extended Multiple Sclerosis Treatment

Yipeng Zhao, Jie Zhang, Xi Cheng, Wenping Huang, Shishi Shen, Shilin Wu, Yiying Huang, Guangjun Nie, Hai Wang,* and Wei Qiu**

This file includes:
Figure S1-S24

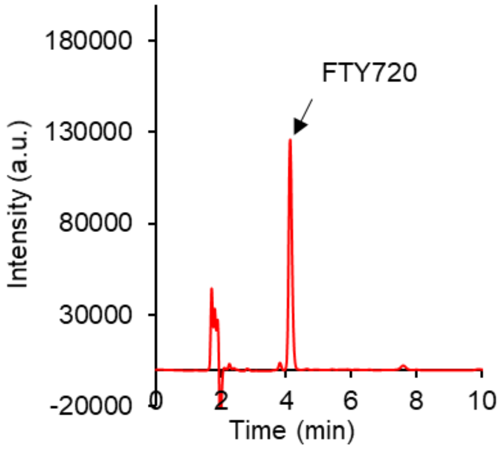

Figure S1. **HPLC analysis of FTY720.** The encapsulation efficiency of FTY720 was determined by HPLC. Arrow indicates the peak of FTY720.

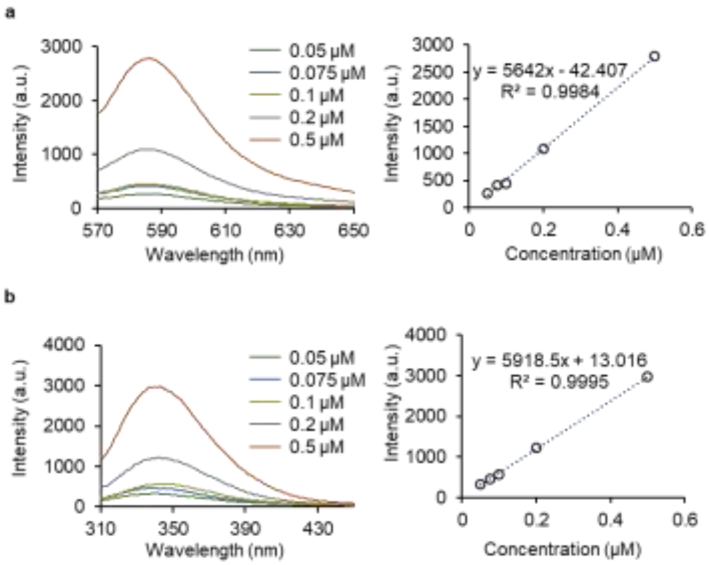

Figure S2. **Fluorescence analysis of L-selectin targeting aptamer and CD47-pHLIP peptide.** (**a**) Fluorescence intensities of L-selectin targeting aptamers at different concentrations. (**b**) Fluorescence intensities of CD47-pHLIP peptide at different concentrations.

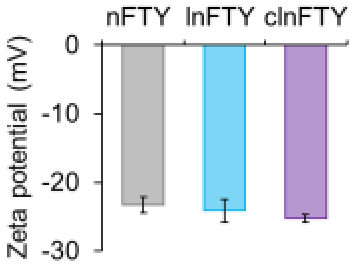

Figure S3. **Zeta potential analysis of nFTY, lnFTY, and clnFTY nanoparticles.** Zeta potentials of nFTY, lnFTY and clnFTY nanoparticles were determined by DLS at room temperature.

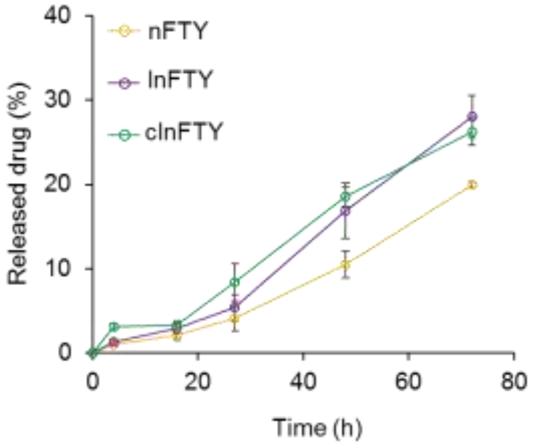

Figure S4. ***In vitro*** **drug release profile of nFTY, lnFTY, and clnFTY.**

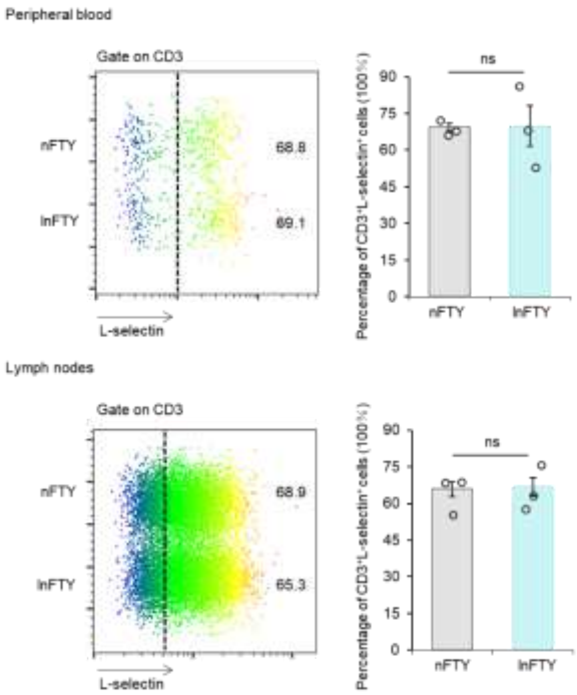

Figure S5. L-selectin $^{+}$ T cells in nanoparticles treated mice. The percentage of L-selectin^{+}CD3^{+} T cells in peripheral blood and LNs after treated with mice nFTY or lnFTY nanoparticles were measured by flow cytometry. Data are presented as mean ± standard error of the (SEM, n = 3), and statistical significance was assessed by the unpaired two-tailed Student's *t*-test.

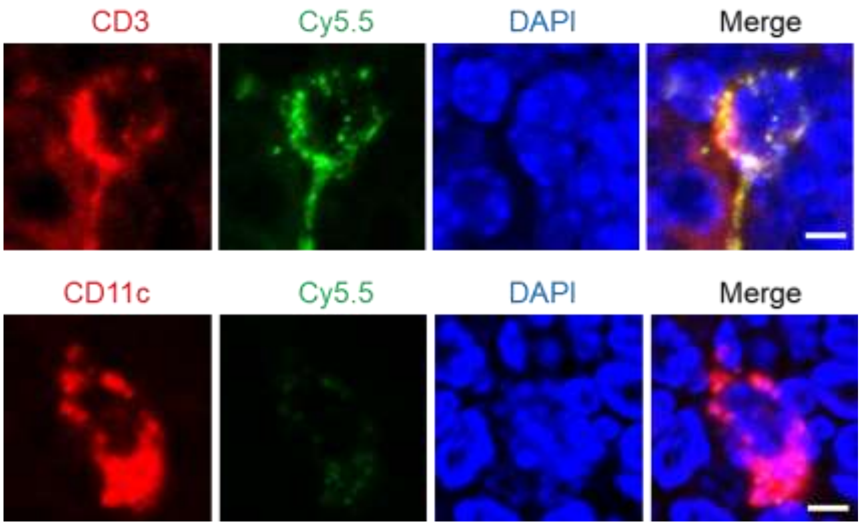

Figure S6. **Distribution of Cy5.5-labeled lnFTY nanoparticles in LNs.** Confocal images of T cells and DCs in the LNs from mice treated with Cy5.5-labeled lnFTY nanoparticles for 24 h. Scar bar, 5μm.

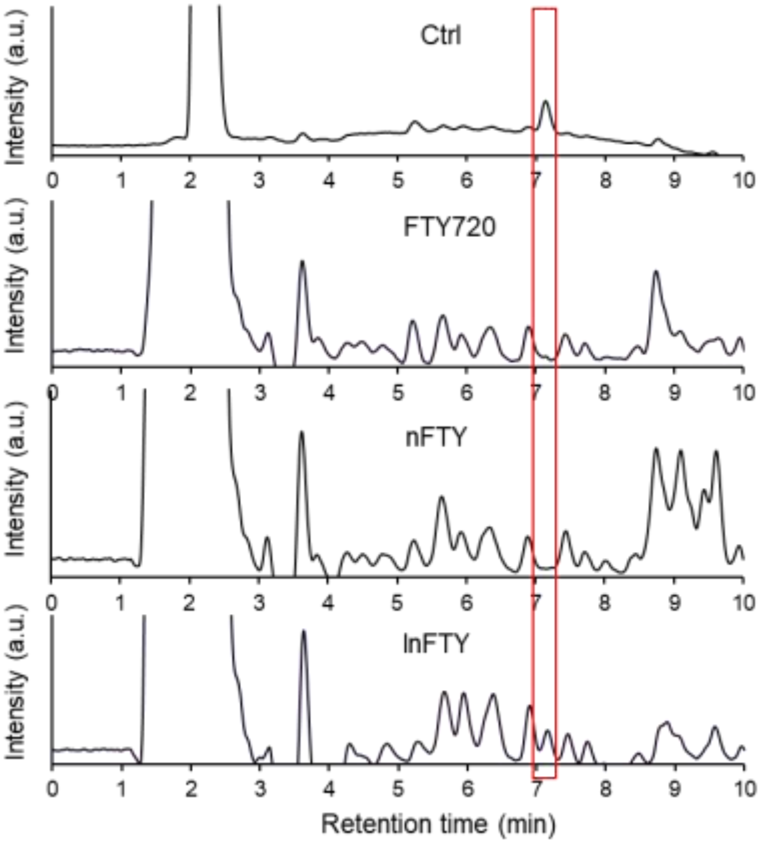

Figure S7. **Detection of FTY720 in LNs.** HPLC data of FTY720 in LNs from mice treated with free FTY720, nFTY, or lnFTY nanoparticles (1mg/kg, FTY720). The solution of FTY720 was used as control. Red box showing the special peak assigned to FTY720.

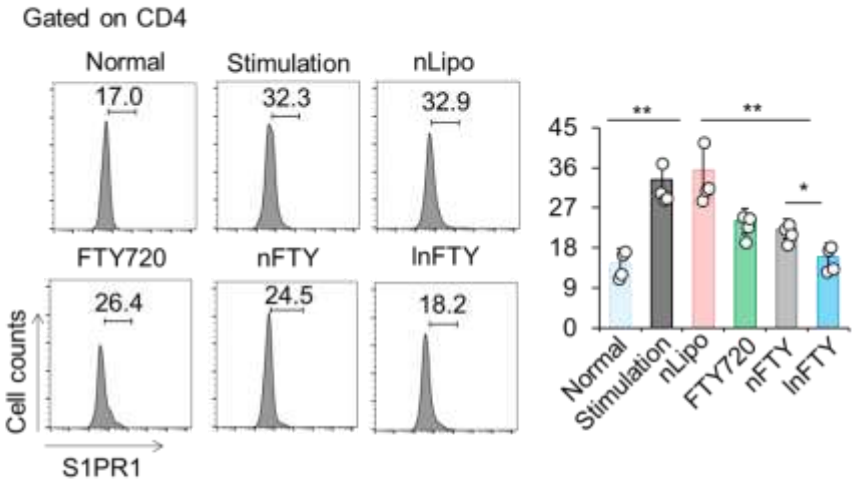

Figure S8. **Inhibition of S1PR1 expression in CD4+ T cells.** The proportion of S1PR1+CD4+ T cells treated with nLipo, free FTY720, nFTY or lnFTY nanoparticles after CD3 and CD28 stimulation was measured by flow cytometry. Data are presented as mean ± standard error of the mean ± SEM (n = 4), and statistical significance was assessed by ANOVA with the Newman-Keuls test. $*p < 0.05$, $**p < 0.01$.

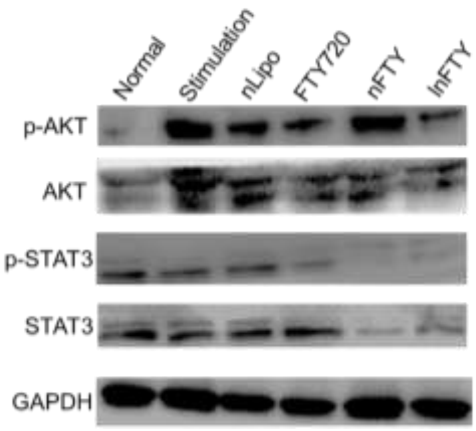

Figure S9. **Analysis of the AKT/STAT3 signaling pathway in CD4+ T cells.** Western blot data of p-AKT and p-STAT3 in CD4+ T cells treated with nLipo, free FTY720, nFTY, or lnFTY nanoparticles following CD3 and CD28 stimulation.

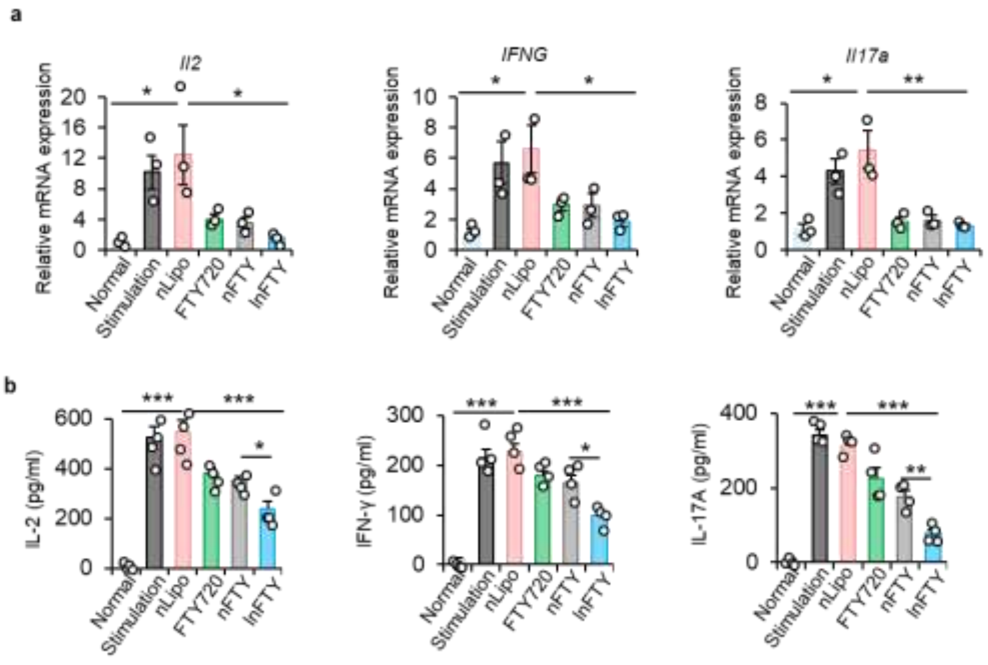

Figure S10. **Analysis of inflammatory cytokine expression in CD4$^+$ T cells.** (**a**) Gene expression of *Il2*, *IFNG* and *Il17a* and (**b**) secretion of IL-2, IFN-γ, and IL-17A in CD4$^+$ T cells treated with nLipo, free FTY720, nFTY or lnFTY nanoparticles after CD3 and CD28 stimulation. Data are presented as mean ± standard error of the mean (SEM) (n = 4), and statistical significance was assessed by ANOVA with the Newman-Keuls test. *$p < 0.05$, **$p < 0.01$, ***$p < 0.001$.

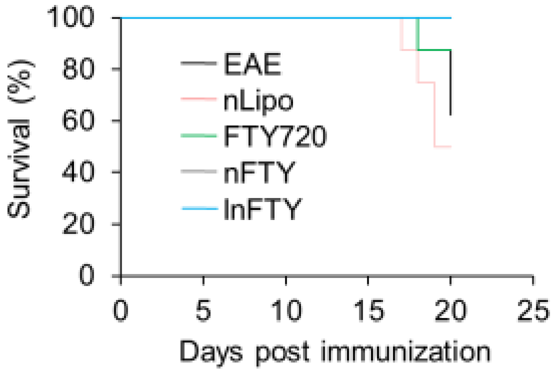

Figure S11. **Survival data of EAE mice with various treatments.**

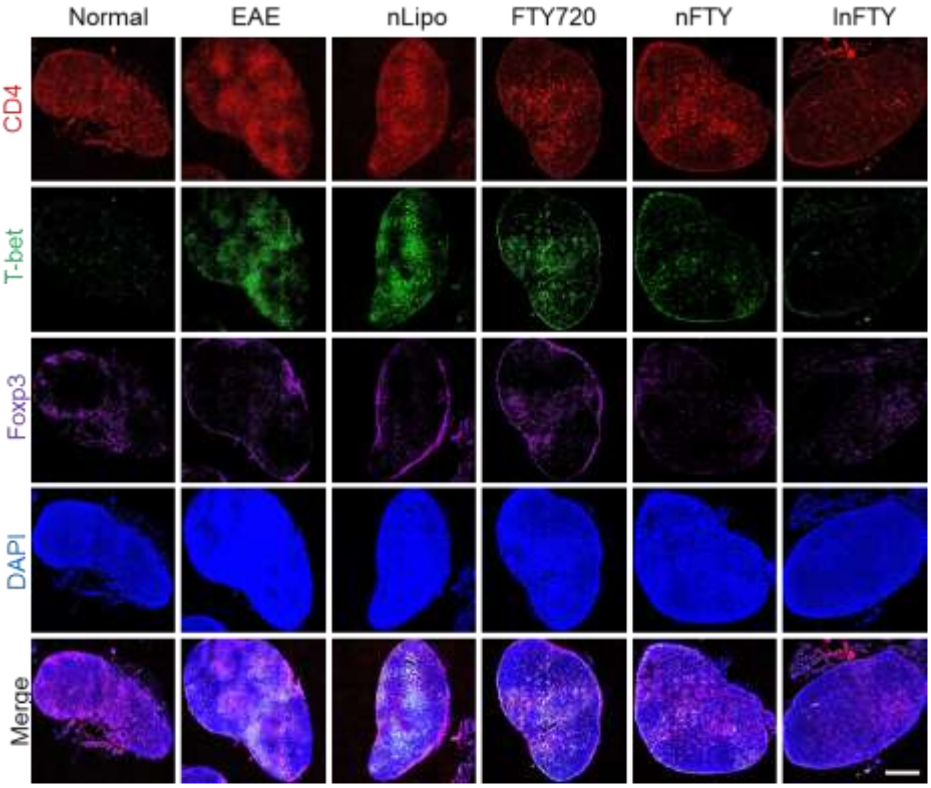

Figure S12. **The expressions of T-bet and Foxp3 in LNs.** Immunofluorescence images of T-bet, Foxp3, and CD4 in LNs from normal mice and EAE mice treated with nLipo, free FTY720, nFTY, or lnFTY nanoparticles. Scale bar, 100 µm.

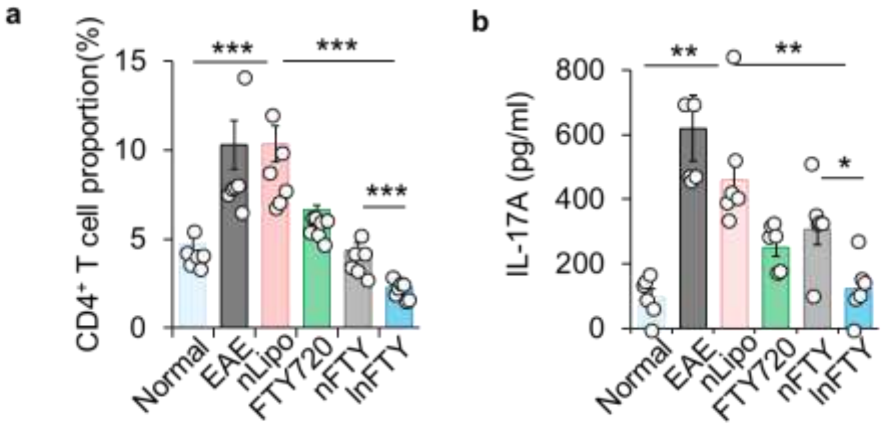

Figure S13. **The proportion of CD4$^+$ T cells and secretion of IL-17A in peripheral blood.** (**a**) The proportion of CD4$^+$ T cells in the peripheral blood of EAE mice treated with nLipo, free FTY720, nFTY, or lnFTY nanoparticles. (**b**) The detection of IL-17A in the serum of EAE mice receiving various treatments. Data are presented as mean ± standard error of the mean (SEM) (n = 6), and statistical significance was assessed by ANOVA with the Newman-Keuls test. *$p < 0.05$, **$p < 0.01$, ***$p < 0.001$.

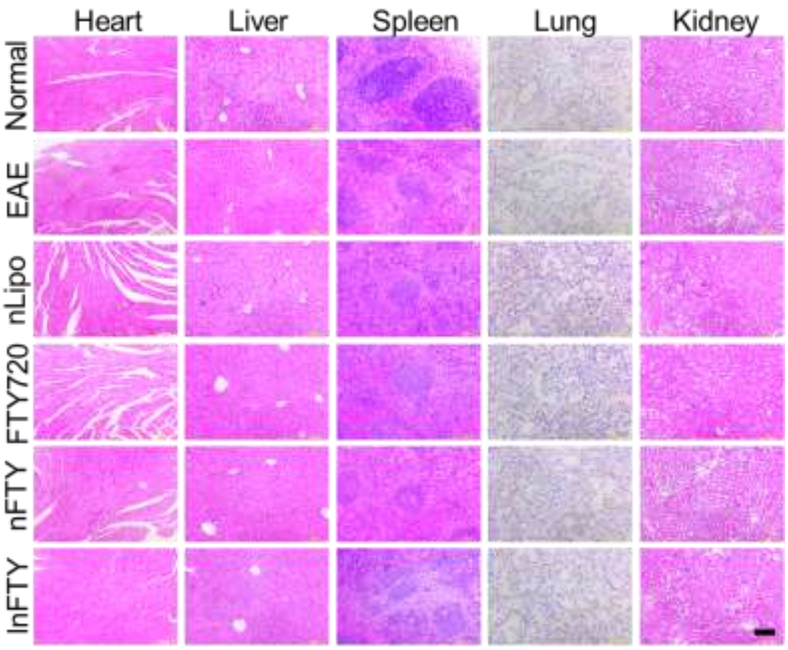

Figure S14. **H&E staining of hearts, livers, spleens, lungs, and kidneys from EAE mice treated with nLipo, free FTY720, nFTY or lnFTY nanoparticles**. H&E images show no apparent toxicity in EAE mice following various treatments. Scale bars, 50 µm.

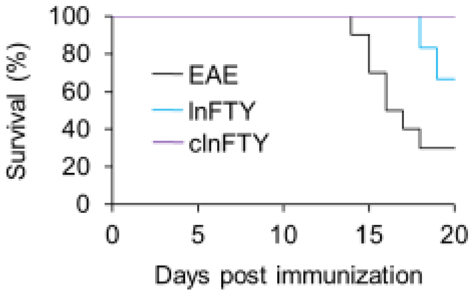

Figure S15. **Survival data of EAE HS mice receiving lnFTY and clnFTY nanoparticles.**

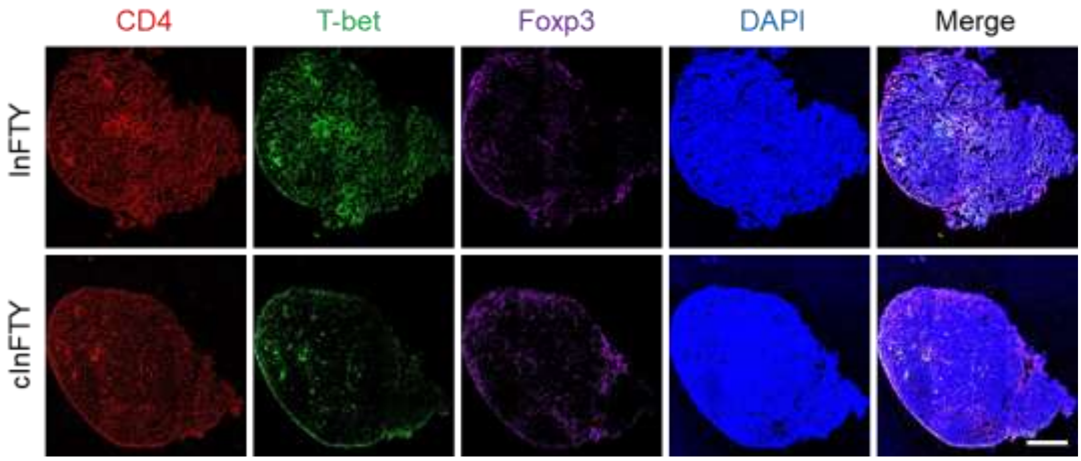

Figure S16. **The expressions of T-bet and Foxp3 in LNs.** Immunofluorescence images of T-bet, Foxp3, and CD4 in LNs from HS mice treated with lnFTY or clnFTY nanoparticles. Scale bar, 100 µm.

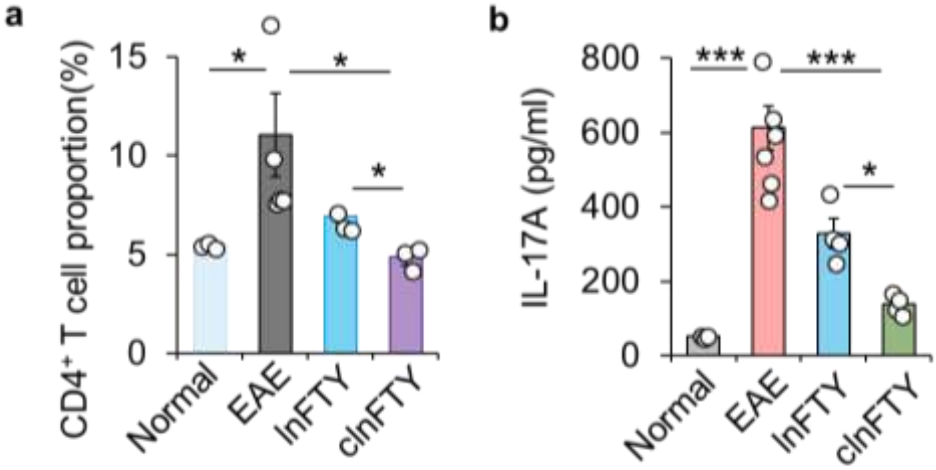

Figure S17. **The proportion of CD4$^+$ T cells and secretion of IL-17A in peripheral blood.** (**a**) The proportion of CD4$^+$ T cells in the peripheral blood of EAE mice treated with lnFTY and clnFTY nanoparticles. (**b**) The detection of IL-17A in the serum of EAE mice receiving various treatments. Data are presented as mean ± standard error of the mean (SEM) (n = 3 for normal, n = 5 for EAE, n = 4 for the lnFTY and clnFTY groups), and statistical significance was assessed by ANOVA with the Newman-Keuls test. *$p < 0.05$, ***$p < 0.001$.

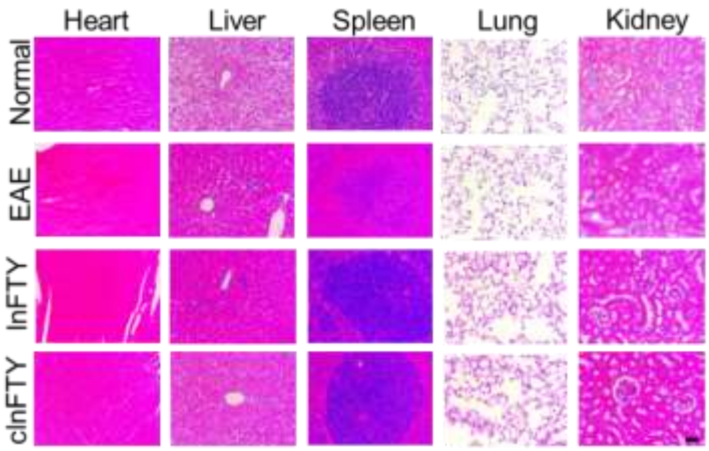

Figure S18. **H&E staining of hearts, livers, spleens, lungs, and kidneys from EAE mice treated with lnFTY and clnFTY nanoparticles**. H&E images show no apparent toxicity in EAE mice following various treatments. Scale bars, 50 µm.

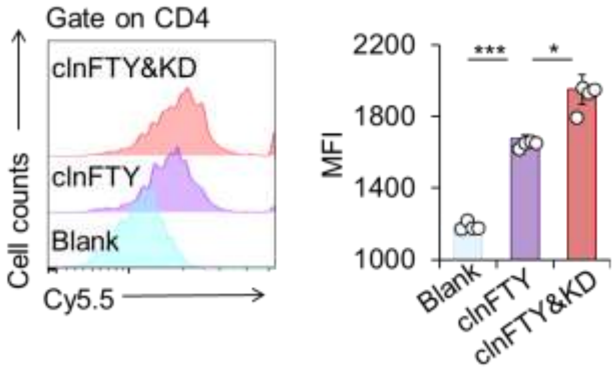

Figure S19. **Ketogenic diet enhancing cellular uptake of clnFTY in CD4$^+$ T cells.** Flow cytometry and statistical analysis showing intracellular fluorescence of Cy5.5-labeled clnFTY in CD4$^+$ T cells in a normal or ketogenic diet medium. Data are presented as mean ± standard error of the mean (SEM) (n = 4), and statistical significance was assessed by ANOVA with the Newman-Keuls test. *$p < 0.05$, ***$p < 0.001$.

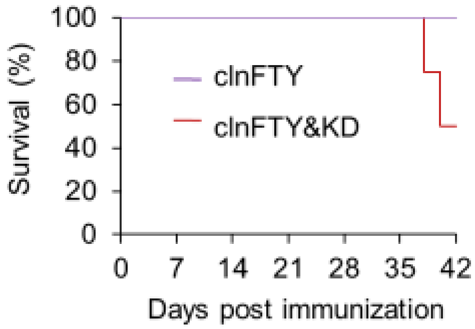

Figure S20. **Survival data of EAE HS mice receiving clnFTY and clnFTY&KD treatments.**

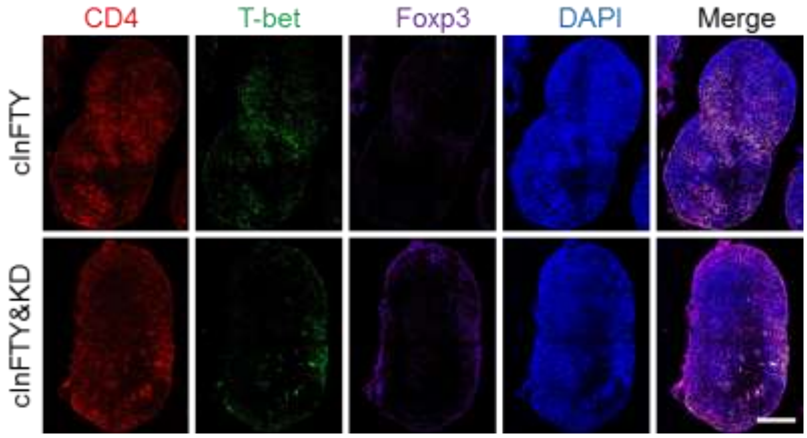

Figure S21. **The expression of T-bet and Foxp3 in LNs.** Immunofluorescence images of T-bet, Foxp3, and CD4 in LNs from HS mice with clnFTY and clnFTY&KD treatments. Scale bar, 100 μm.

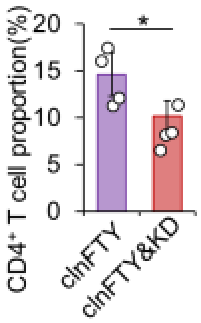

Figure S22. **Detection of CD4$^+$ T cells in peripheral blood.** The proportion of CD4$^+$ T cells in the peripheral blood of EAE HS mice treated with clnFTY or clnFTY&KD. Data are presented as mean ± standard error of the mean (SEM) (n = 4), and statistical significance was assessed by the unpaired two-tailed Student's t-test. *$p < 0.05$.

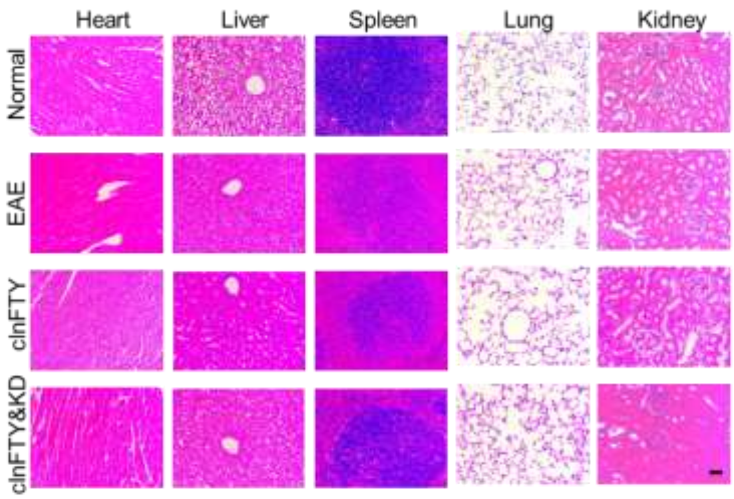

Figure S23. **H&E staining of hearts, livers, spleens, lungs, and kidneys from EAE HS mice treated with clnFTY and clnFTY&KD**. H&E images show no apparent toxicity in EAE HS mice following various treatments. Scale bars, 50 µm.

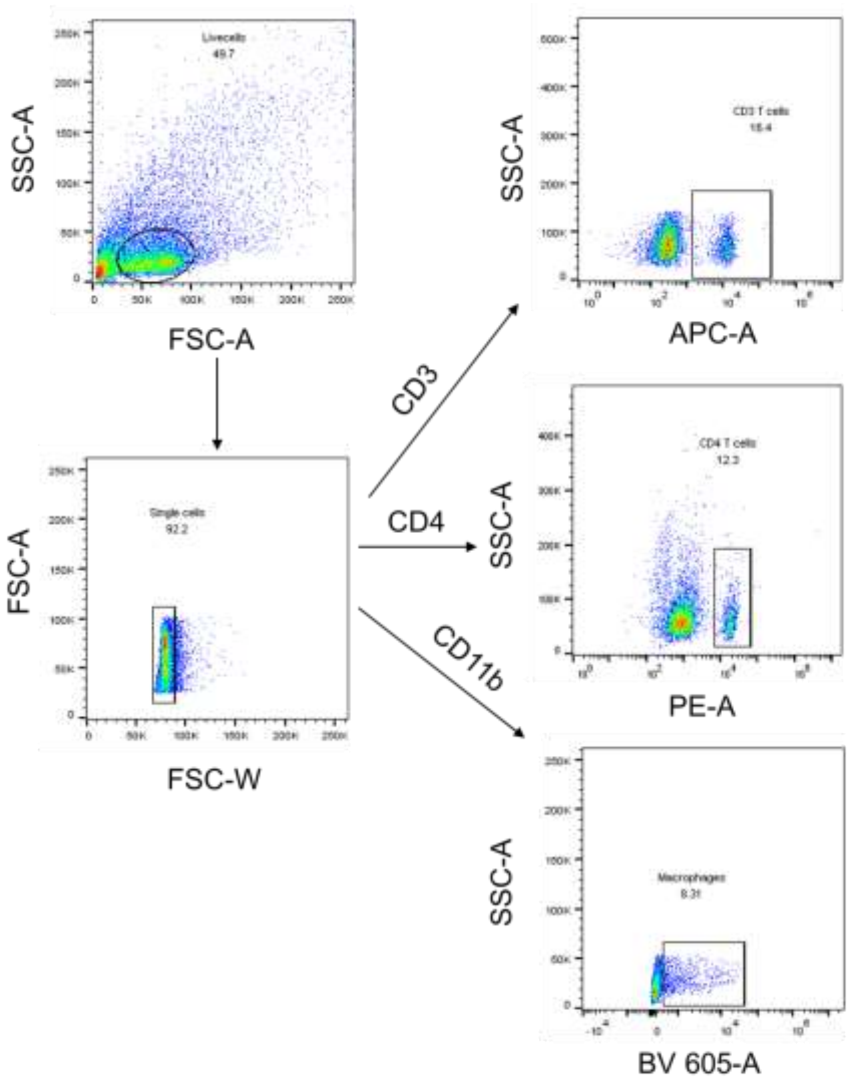

Figure S24. **Gating strategy for FACS cell sorting**. Live cells were first gated based on FSC-A/SSC-A, as shown in the upper left; single cells were then gated based on FSC-A/FSC-W, as shown in the bottom left; CD3s were gated based on APC-A/SSC-A, CD4s were gated based on PE-A/SSC-A and CD11bs were gated based on BV 605-A/SSC-A as shown in the right panel.

Uncropped blots in Figure S9

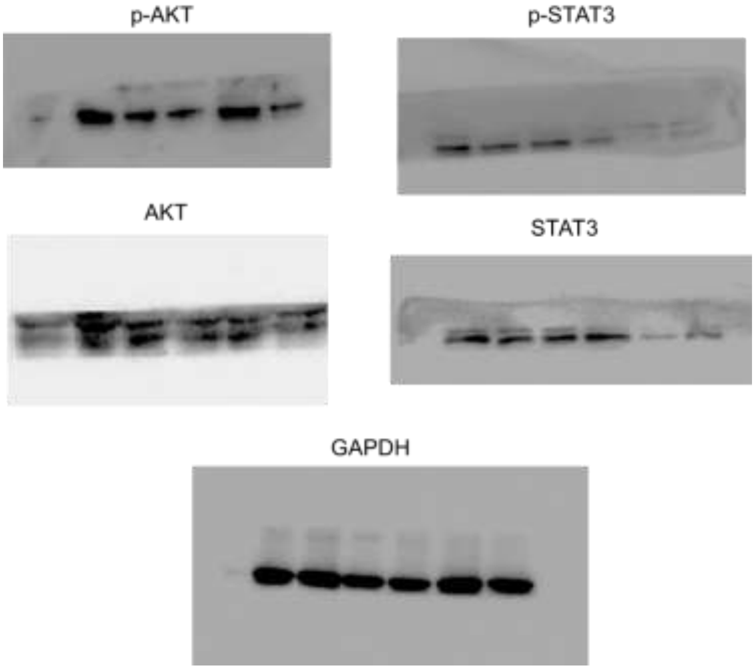

Supplement: Supplementary file 1 — Supporting Information [file ADVS-10-2300738-s001.pdf]
